# Inhibition of β-Catenin signaling suppresses pancreatic tumor growth by disrupting nuclear β-Catenin/TCF-1 complex: Critical role of STAT-3

**DOI:** 10.18632/oncotarget.3427

**Published:** 2015-03-21

**Authors:** Kartick C. Pramanik, Neel M. Fofaria, Parul Gupta, Alok Ranjan, Sung-Hoon Kim, Sanjay K. Srivastava

**Affiliations:** ^1^ Department of Biomedical Sciences and Cancer Biology Center, Texas Tech University Health Sciences Center, Amarillo, TX 79106, USA; ^2^ Cancer Preventive Material Development Research Center, College of Korean Medicine, Department of Pathology, Kyunghee University, Dongdaemun-ku, Seoul 131–701, South Korea

**Keywords:** STAT3, β-Catenin, GSK-3β, pancreatic cancer, orthotopic tumor

## Abstract

Aberrant activation of β-catenin/TCF signaling is related to the invasiveness of pancreatic cancer. In the present study, we evaluated the effect of capsaicin on β-catenin/TCF signaling. In a concentration and time-dependent study, we observed that capsaicin treatment inhibits the activation of dishevelled (Dsh) protein DvI-1 in L3.6PL, PanC-1 and MiaPaCa-2 pancreatic cancer cells. Capsaicin treatment induced GSK-3β by inhibiting its phosphorylation and further activated APC and Axin multicomplex, leading to the proteasomal degradation of β-catenin. Expression of TCF-1 and β-catenin-responsive proteins, c-Myc and cyclin D1 also decreased in response to capsaicin treatment. Pre-treatment of cells with MG-132 blocked capsaicin-mediated proteasomal degradation of β-catenin. To establish the involvement of β-catenin in capsaicin-induced apoptosis, cells were treated with LiCl or SB415286, inhibitors of GSK-3β. Our results reveal that capsaicin treatment suppressed LiCl or SB415286-mediated activation of β-catenin signaling. Our results further showed that capsaicin blocked nuclear translocation of β-catenin, TCF-1 and p-STAT-3 (Tyr705). The immunoprecipitation results indicated that capsaicin treatment reduced the interaction of β-catenin and TCF-1 in the nucleus. Moreover, capsaicin treatment significantly decreased the phosphorylation of STAT-3 at Tyr705. Interestingly, STAT-3 over expression or STAT-3 activation by IL-6, significantly increased the levels of β-catenin and attenuated the effects of capsaicin in inhibiting β-catenin signaling. Finally, capsaicin mediated inhibition of orthotopic tumor growth was associated with inhibition of β-catenin/TCF-1 signaling. Taken together, our results suggest that capsaicin-induced apoptosis in pancreatic cancer cells was associated with inhibition of β-catenin signaling due to the dissociation of β-catenin/TCF-1 complex and the process was orchestrated by STAT-3.

## INTRODUCTION

Today pancreatic cancer ranks as the fourth leading cause of cancer related deaths in the United States [1]. In spite of significant advances in surgical care, chemotherapy and radiotherapy, the average five year survival rate of pancreatic cancer is less than 5% [1]. Most of the treatment failures in the clinic are due to the development of resistance to systemic therapy. Therefore, development of novel agents, which can prevent and treat pancreatic cancer is an important mission. β-catenin and other proteins including axin and adenomatous polyposis coli (APC) in the Wnt signaling pathway play an important role in many types of human cancers, including pancreatic cancer [2–4]. Mutation of β-catenin/APC or aberrant activation of Wnt and β-catenin signaling have been reported in pancreatoblastomas [5, 6], acinar cell carcinoma [7], pancreatic ductal adenocarcinoma [3, 8, 9] and solid pseudopapillary neoplasm (SPN) [10–13]. In the presence of Wnt inhibitor, β-catenin is targeted for degradation through phosphorylation of glycogen synthase kinase-3β (GSK-3β), which forms complexes with APC and Axin. However, when Wnt or Frizzled are up-regulated, this complex is inhibited and β-catenin is phosphorylated resulting in its accumulation in the cytoplasm and subsequent nuclear translocation. After nuclear translocation, β-catenin is stabilized and binds to TCF/LEF transcriptional co-activators, resulting in the up-regulation of transcription responsive genes cyclin D1 and c-Myc. Through regulating target gene expression, β-catenin/TCF signaling is involved in sequential neoplastic development from initiation, progression to metastasis [5, 14–16]. Published studies suggest that inhibition of β-catenin decreases TCF transcriptional activity and induces caspase-3-mediated apoptosis [17].

Signal transducer and activator of transcriptional 3 is a member of STAT family present in cytoplasm. Phosphorylated STAT-3s dimerize and translocate to the nucleus, where it regulates the transcription of genes and modulate various physiological functions, including cell cycle regulation, cell survival and angiogenesis [18, 19]. Interestingly, recently published papers suggest that STAT-3 activation might participate in β-catenin nuclear accumulation in colorectal cancer [20].

Capsaicin is a major component of hot chili pepper. Several studies have shown significant chemopreventive and chemotherapeutic effects of capsaicin against certain mutagens and carcinogens [21–26]. Capsaicin has also shown effectiveness in preventing several types of cancer through various mechanisms [27–31]. In the present study, we evaluated the effect of capsaicin in β-catenin and TCF-1 signaling and a possible link to STAT-3.

## RESULTS

### Capsaicin triggers apoptosis by inhibiting β-catenin/TCF-1 signaling in pancreatic cancer cells

**Figure 1:**
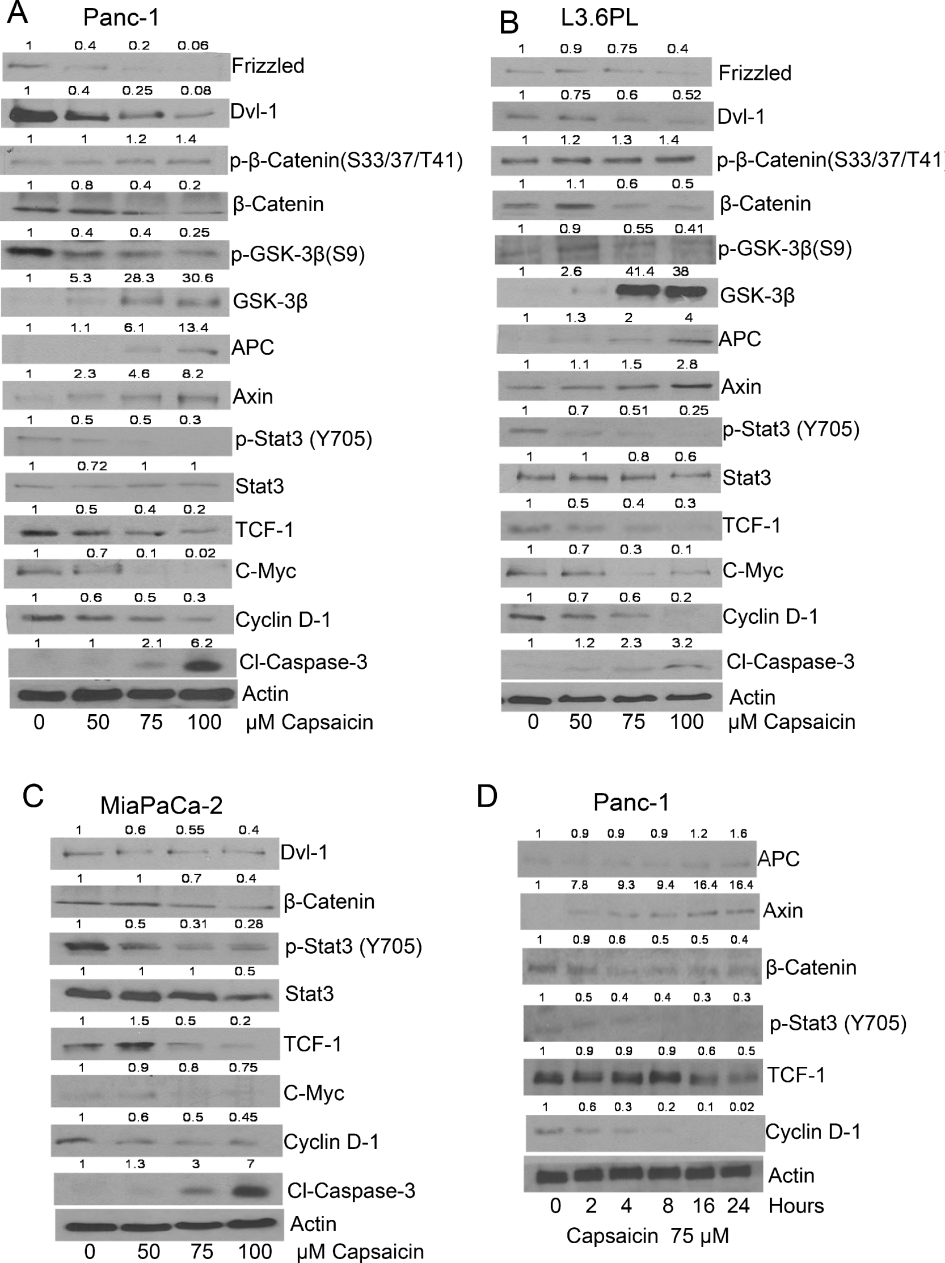
Effect of capsaicin on β-catenin and TCF-1 signaling pathway in pancreatic cancer cells **(A & D)** PanC-1 (B) L3.63PL (C) MiaPaCa-2 cells were treated with various concentrations of capsaicin or at various time points with 75 μM of capsaicin for 24 h. Cells were lysed and subjected to western blot. Immunoblots were probed for Frizzled, DVI-1, p-β-catenin (S33/37/T41), β-catenin, p-GSK-3β (S9), GSK-3β, APC, Axin, p-Stat-3 (Tyr705), Stat-3, TCF-1, c-Myc, cyclin D1and Cl-caspase-3. Same blots were stripped and reprobed for actin to ensure equal protein loading. The experiments were repeated three times with similar results obtained.

In order to determine the effect of capsaicin on β-catenin/TCF-1 signaling, PanC-1, L3.6PL and MiaPaCa-2 cells were treated with various concentrations of capsaicin for 24 h or with 75 μM capsaicin for various time points. Our results revealed that capsaicin treatment decreased the phosphorylation of GSK-3β at S9 and STAT-3 at Y705 and increased the phosphorylation of β-catenin at S33/37/T41 (Figure 1A, 1B & 1C). Capsaicin treatment further reduced the protein levels of Frizzled, DVI-1, β-catenin, TCF-1, c-Myc and Cyclin D1, while increased GSK-3β, APC, and Axin protein levels (Figure 1A, 1B & 1C). These results suggest that capsaicin treatment inhibits the Frizzled receptor, inactivates DSH, resulting in increased APC/Axin/GSK-3β complex formation, leading to the prevention of β-catenin stabilization and activation of β-catenin/TCF-1 mediated transcriptional responsive genes. Further, cleavage of caspase-3 was observed by capsaicin treatment indicating apoptosis. In a time-dependent study, β-catenin levels began decreasing after 4 h of capsaicin treatment concomitant with the increase in APC and Axin levels, indicating that capsaicin mediated inhibition of cytosolic β-catenin increased APC, Axin and GSK-3β complex. Capsaicin treatment also decreased TCF-1 at 16 h and CyclinD1 at 2 h (Figure 1D). However cleavage of caspase-3 and PARP was observed only at 16 h as described in our previous study [32]. Overall, these results suggest that capsaicin-induced apoptosis was associated with the inhibition β-catenin/TCF-1 signaling.

### Capsaicin treatment inhibits nuclear localization of β-catenin, TCF-1 and p-STAT-3(Tyr 705)

**Figure 2:**
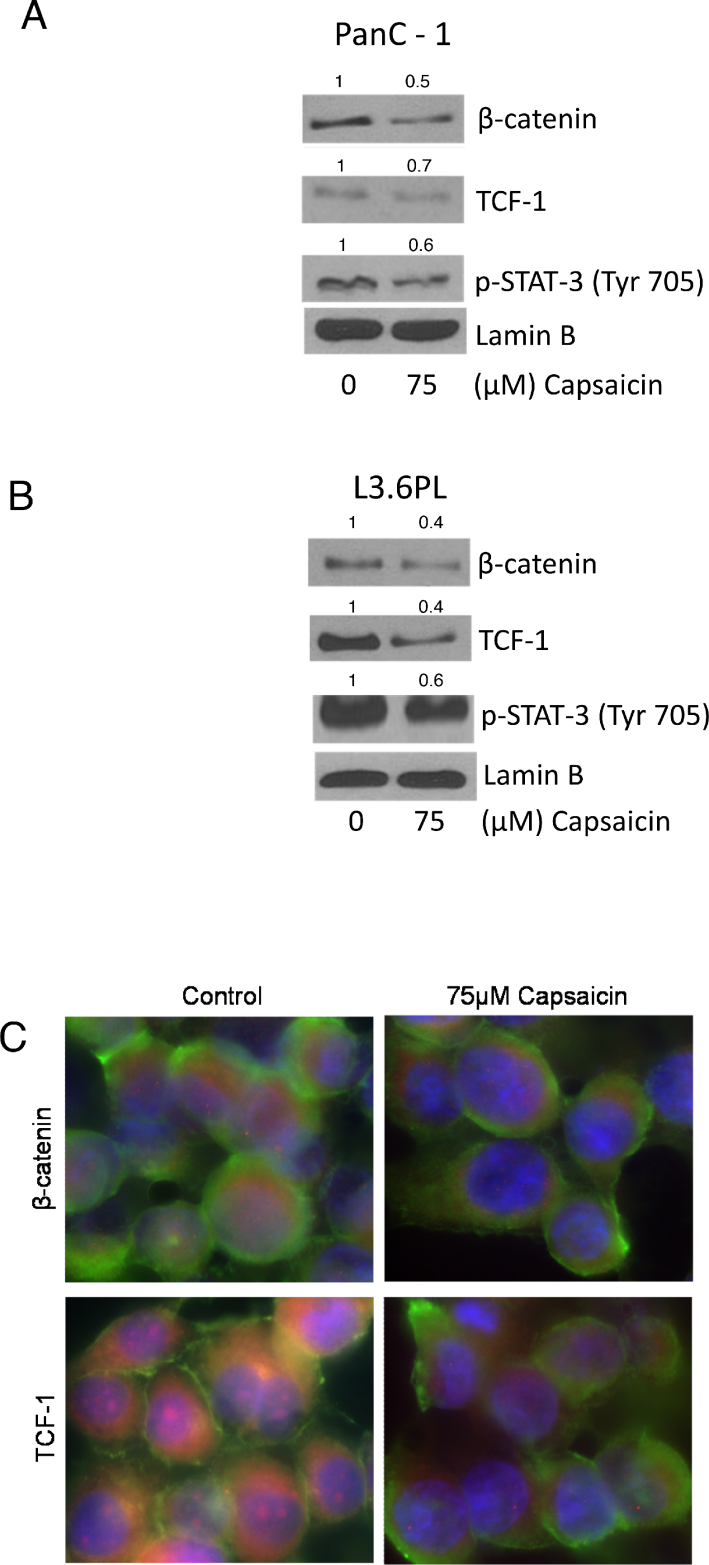
Effect of capsaicin in nuclear localization of β-catenin, TCF-1 and p-Stat-3(Tyr 705) **(A)** PanC-1 and **(B)** L3.6PL cells were treated with 75 μM capsaicin for 24 h and nuclear fraction were isolated using nuclear fractionation kit. Represented western blots were immunoblotted with β-catenin, TCF-1 and p-Stat-3 (Tyr705) antibodies. The same blots were then stripped and reprobed with lamin B to ensure equal protein loading. **(C)** PanC-1 cells were treated with DMSO or 75 μM capsaicin, immunostained with β-catenin and TCF-1 (red), and actin (green) antibodies, then visualized under fluorescence microscope (Olympus Inc.). The experiments were repeated three times with similar results obtained.

β-catenin upon translocation to the nucleus associates with transcription factor TCF and activates β-catenin/TCF-1 complex leading to the transcription of downstream gene such as c-Myc and cyclin D1, hence increase in cell proliferation. Since we observed decrease in c-Myc and cyclinD1 expression by capsaicin treatment, we next wanted to see β-catenin and TCF-1 protein levels in the nucleus. Our results show that β-catenin and TCF-1 levels decreased significantly in the nuclear fraction of capsaicin treated PanC-1 and L3.6PL cells (Figure 2A & 2B). STAT-3 upon phosphorylation dimerizes and translocates to nucleus to transcribe cell survival genes. Our results clearly demonstrate that capsaicin treatment blocks nuclear entry of p-STAT-3 (Tyr705) in both the cell lines (Figure 2A & 2B). Nuclear localization of β-catenin and TCF-1 were further confirmed by immunofluorescence. Capsaicin treatment decreased immuno-staining of β-catenin and TCF-1 in the nucleus (Figure 2C), suggesting that capsaicin treatment inhibits nuclear translocation of β-catenin and thereby inhibits β-catenin and TCF-1 mediated transcription of responsive genes.

### Capsaicin treatment disrupts β-catenin-TCF-1 complex in nucleus

**Figure 3:**
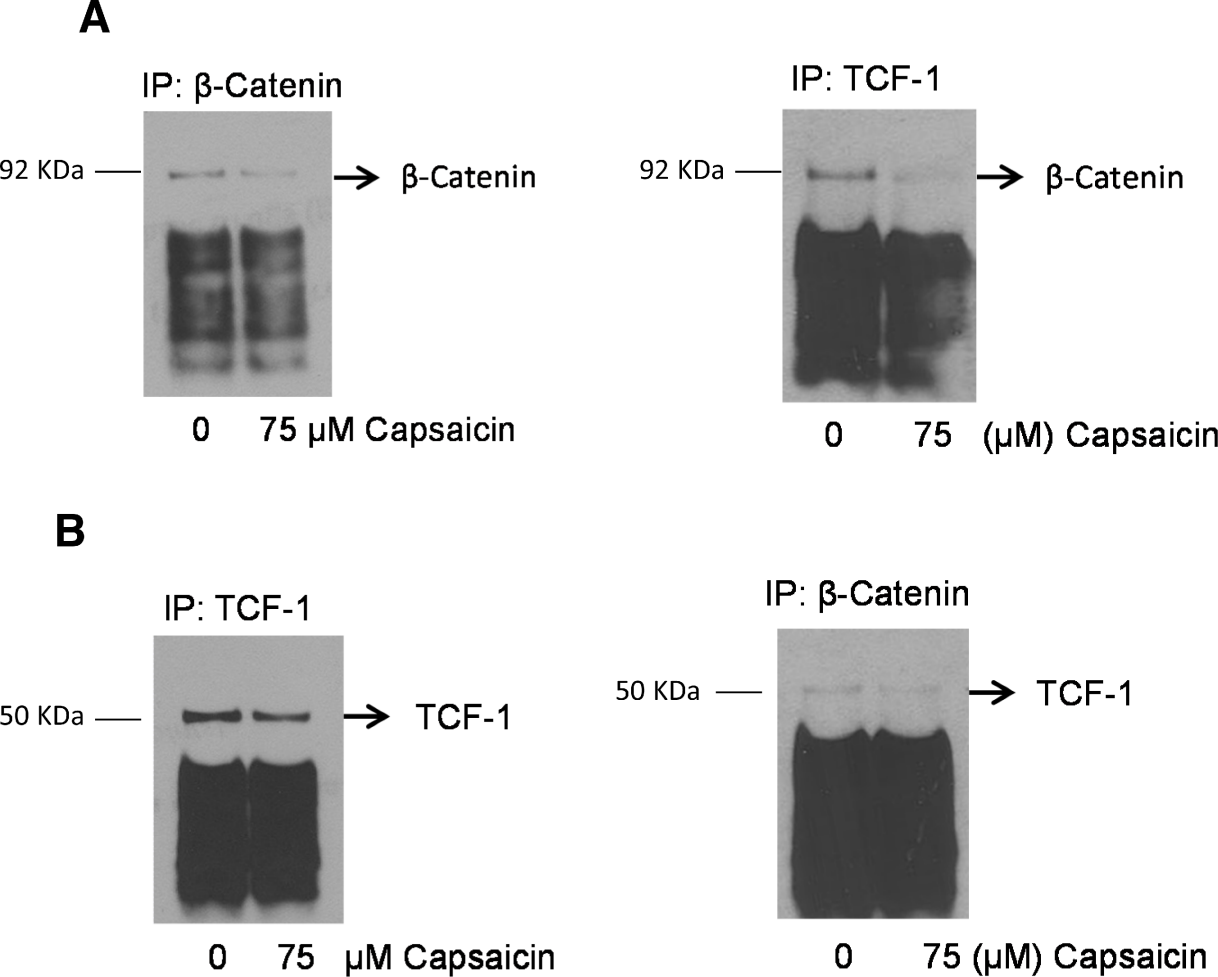
Capsaicin treatment disrupts nuclear β-catenin and TCF-1 complex PanC-1 cells were treated with DMSO or 75 μM capsaicin for 24 h and nuclear fraction was isolated by nuclear fractionation kit and immunoprecipitated with **(A** & **B)** β-catenin and TCF-1 and immunobloted with β-catenin and TCF-1 antibodies respectively. The experiments were repeated three times with similar results obtained.

We next wanted to see whether capsaicin disrupts the β-catenin/TCF-1 complex in the nucleus. In order to do that, PanC-1 cells were treated with 75 μM capsaicin for 24 h and nuclear fraction was isolated using nuclear extraction kit before being subjected to immunoprecipitation assay. β catenin and TCF-1 were immuno-precipitated and immuno-blotted with their respective antibodies. Our co-immunoprecipitation assay from nuclear fraction showed that capsaicin treatment inhibits β-catenin levels when immunoprecipated with β-catenin or TCF-1 antibodies. On the other hand, capsaicin treatment decreased TCF-1 levels when immunoprecipitated with TCF-1 or β-catenin antibodies, indicating capsaicin treatment disrupts association of β-catenin/TCF-1complex in the nucleus (Figure 3A & 3B).

### Capsaicin increases proteasomal degradation of β-catenin

**Figure 4:**
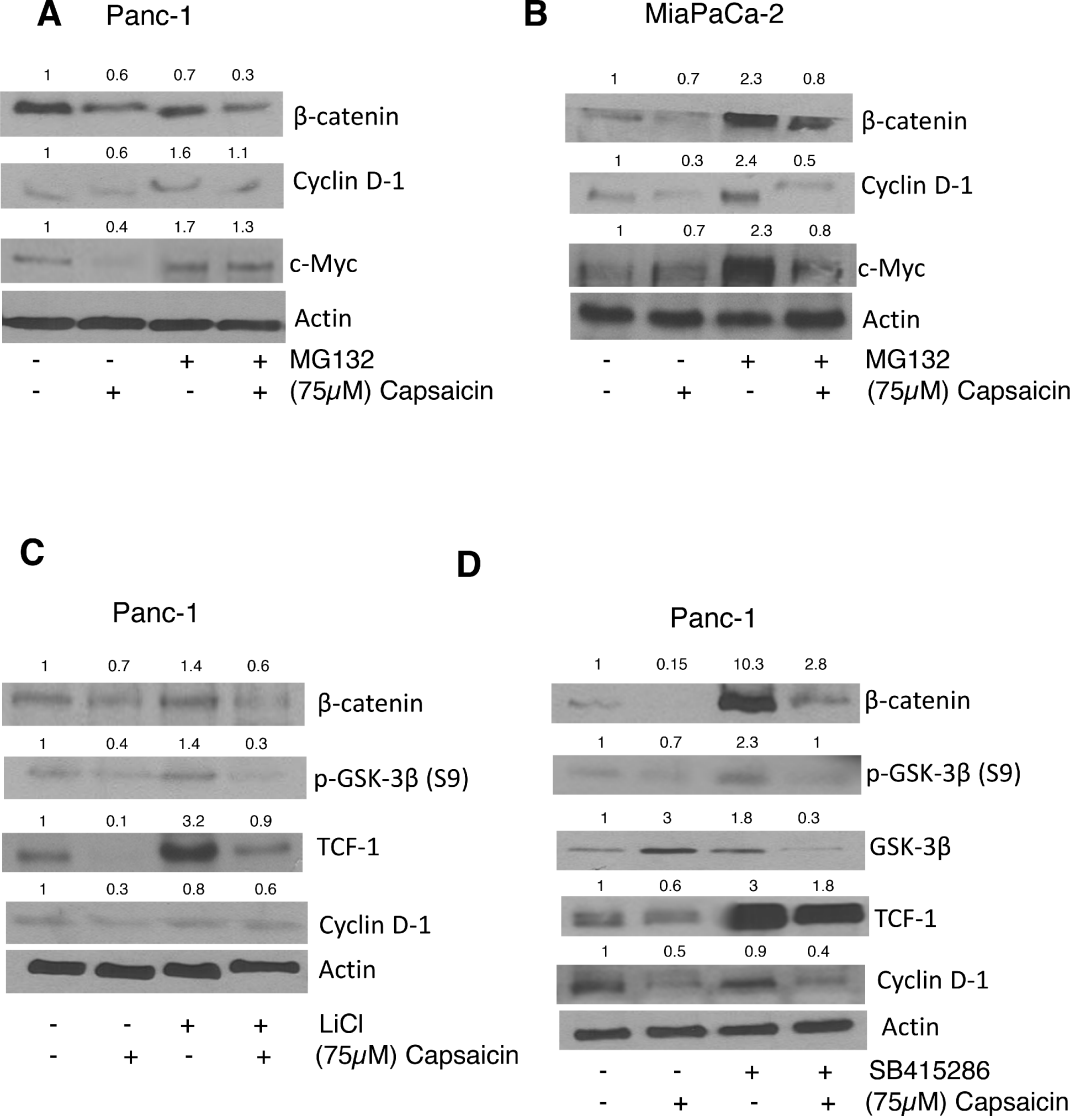
Effect of capsaicin on MG132, Licl and SB415286 treatment **(A)** PanC-1 and **(B)** MiaPaCa-2 cells were pre-treated with 10 μM MG-132 followed by treatment with 75 μM capsaicin for 24 h. After which whole cell lysates were immunoblotted with β-catenin, cyclin D1, c-Myc (C&D) in a separate experiment, PanC-1 cells were pre-treated with 40 mM Licl or 50 μM SB415286, followed by 75 μM capsaicin treatment for 24 h and then whole cell lysates were immunoblotted with β-catenin, p-GSK-3β, GSK-3β, TCF-1, Cyclin D1. The same blot was stripped and reprobed for actin to ensure equal protein loading.

Inhibition of proteasome-mediated proteolysis stabilizes β-catenin leading to its accumulation in the nucleus. Thus, we wanted to see whether blocking proteosomal degradation of β-catenin with MG-132 could increase β-catenin levels. Pre-treatment of cells with MG-132 inhibited the proteosomal degradation of β-catenin, which in turn increased the expression of downstream molecules such as cyclin D1 and c-Myc in PanC-1 and MiaPaCa-2 cells (Figure 4A & 4B), suggesting that capsaicin treatment increases proteosomal degradation of β-catenin and thereby inhibits β-catenin/TCF-1 signaling in pancreatic cancer cells.

### Capsaicin treatment suppresses LiCl/SB415286 mediated activation of β-catenin signaling

Activation of Wnt/β-catenin signaling inhibits APC/Axin/GSk-3β complex, which prevents phosphorylation of β-catenin leading to its translocation and accumulation in the nucleus. We therefore wanted to see whether GSK-3β inhibitor LiCl or SB415286 could block capsaicin mediated inhibition of β-catenin signaling. To do so, PanC-1 cells were pretreated with LiCl or SB415286 for 1 h, followed by 75 μM capsaicin treatment for 24 h. As expected, LiCl and SB415286 activated β-catenin/TCF-1 signaling by increasing β-catenin, p-GSK-3β and TCF-1 levels (Figure 4C & 4D). However, these effects were abrogated by capsaicin treatment indicating that capsaicin treatment inhibits β-catenin and TCF-1 signaling and thereby inhibits pancreatic cancer (Figure 4C & 4D).

### Capsaicin suppresses β-catenin/TCF-1 signaling through STAT-3

**Figure 5:**
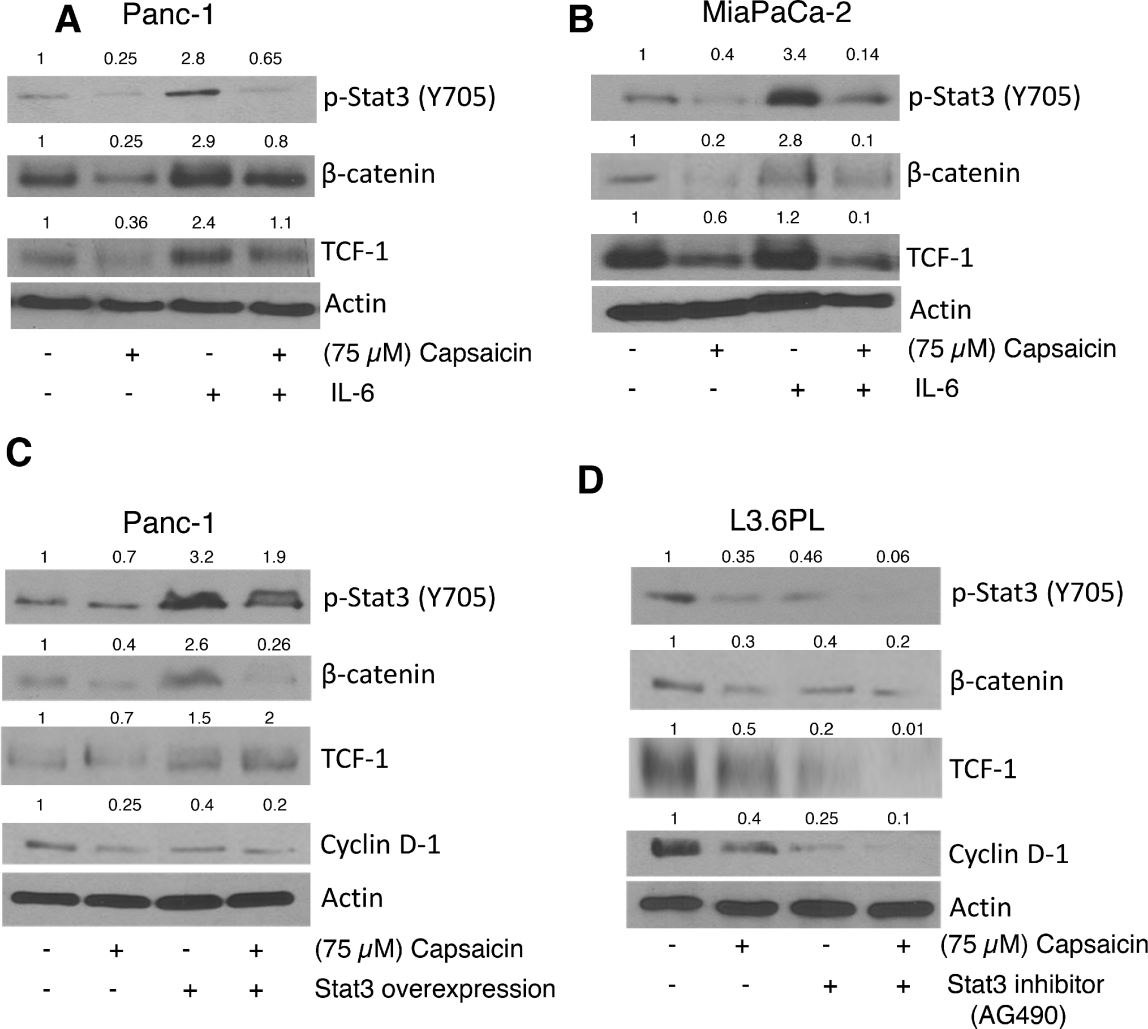
Effect of capsaicin on IL-6, Stat-3 inhibitor and Stat-3 over-expression plasmid **(A)** PanC-1 **(B)** MiaPaCa-2 cells were Pre-treated with 20 ng/ml IL-6 followed by 75 μM capsaicin treatment for 24 h. **(C)** PanC-1 cells were transiently transfected with 2 μg of Stat-3 plasmid using FuGENE transfection reagent for 24 h and then transfected cells were treated with 75 μM capsaicin for 24 h. **(D)** L3.6PL cell were pre-treated with 10 μM Stat-3 inhibitor, followed by 75 μM capsaicin treatment for 24 h. Whole cell lysates were immunobloted with p-Stat-3 (Tyr 705), β-catenin, TCF-1, Cyclin D1. The same blots were stripped and reprobed for actin to ensure equal protein loading.

STAT-3 plays a critical role in Wnt/β-catenin signaling. We wanted to see whether activation of STAT-3 by IL-6 or overexpression of STAT-3 by transient transfection increases β-catenin/TCF-1 signaling. Cells were pre-treated with IL-6 followed by 75 μM capsaicin treatment for 24 h. Our results revealed that activation of STAT-3 by IL-6 increased β-catenin and TCF-1 levels, but attenuated by capsaicin treatment (Figure 5A & 5B). To confirm this hypothesis, cells were transiently transfected with STAT-3 plasmid and treated with 75 μM capsaicin for 24 h. Our results demonstrate that over-expression of STAT-3 increased β-catenin levels. Nonetheless, capsaicin treatment suppressed these effects (Figure 5C). To further confirm this phenomenon, cells were pretreated with STAT-3 inhibitor followed by 24 h capsaicin treatment. Our results show that STAT-3 inhibitor increased capsaicin mediated suppression of β-catenin/TCF-1 signaling, suggesting a regulatory role of STAT-3 in β-catenin/TCF-1 signaling in pancreatic cancer cells (Figure 5D).

### Capsaicin inhibits the growth of orthotropic pancreatic tumor by inhibiting β-catenin/TCF-1 signaling

**Figure 6:**
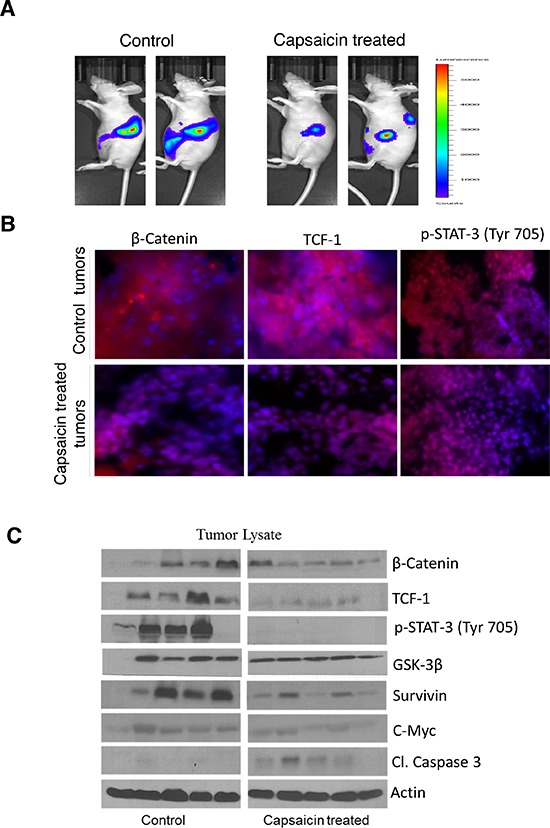
Capsaicin inhibits the growth of orthotropic pancreatic tumor by inhibiting β-catenin/TCF-1 signaling In the present study we used our previous orthotopic experiment tumors to represent the following parameters. **(A)** Around 1 × 10^6^ PanC-1-luc cells were injected orthotopically in the pancreas with minor surgery. Once mice had stable image, animals were randomly divided into two groups. The treated group received 5 mg/kg body weight capsaicin by oral gavage every day, whereas control group received vehicle only. Animals were imaged using IVIS Bio Luminescent System. Representative images of control and capsaicin treated mice are shown in Figure 6A. **(B)** Tumors from control and capsaicin treated mice were dissected out and kept in 4% formalin solution. Tumors were then sliced about 10 μm thick and placed on glass slides and kept in frozen for 24 h. Treated and untreated tumors were immunostained with β-catenin (red), TCF-1(red) and p-Stat-3 (Tyr 705) (red) antibodies and visualized under fluorescence microscope (Olympus Inc.). The experiments were repeated three times with similar results obtained. In order to determine the mechanism of tumor growth suppression, tumors were homogenized, lysed and subjected to western blot. **(C)** Representative immunnoblots showed the effect of capsaicin treatment on phosphorylation of p-Stat-3 (Tyr 705) and protein levels of β-catenin, TCF-1, GSK-3β, Survivin, c-Myc and Cl-caspase-3. Each band represents tumor from different mouse. The blots were stripped and reprobed for actin to ensure equal protein loading.

In order to determine whether oral administration of capsaicin can suppress the growth of pancreatic tumors, we orthotopically implanted Panc1-Luc pancreatic tumor cells in athymic nude mice [32]. After three week of tumor cell implantation, animals were fed 5 mg/kg capsaicin every day by oral gavage. Non-invasive imaging of mice revealed that capsaicin treatment substantially suppressed the growth of pancreatic tumors as shown in Figure 6A [32]. To confirm that the tumor growth suppressive effects of capsaicin were associated with inhibition of β-catenin, TCF-1 and p-STAT-3 (Tyr 705), tumors were dissected out and subjected to immunofluorescence (Figure 6B). Our results revealed that tumors from capsaicin treated mice exhibited decreased red staining for β-catenin, TCF-1 and p-STAT-3 (Tyr 705), indicating that capsaicin treatment inhibits β-catenin, TCF-1 and p-STAT-3 (Tyr 705) *in vivo*. In order to further confirm that capsaicin mediated tumor growth suppression was associated with inhibition of β-catenin/TCF-1 signaling, tumors from control and capsaicin-treated mice were subjected to western blotting. Our results demonstrate that capsaicin treatment inhibited β-catenin, TCF-1, p-STAT-3, survivin and c-Myc levels whereas increased cleavage of caspase-3 in the tumors of capsaicin treated mice as compared to controls (Figure 6C). These results clearly indicate that tumor growth suppression by capsaicin was indeed associated with the inhibition of β-catenin/TCF-1 signaling *in vivo* (Figure 6C).

## DISCUSSION

Pancreatic cancer is one of the leading causes of cancer-related deaths worldwide, therefore, an effective treatment approach is required for controlling this malignancy. We have previously demonstrated that capsaicin-mediated inhibition of pancreatic cancer cells was associated with ROS generation and dissociation of ASK1 and Trx-1complex [32, 33]. β-catenin and other components of Wnt signaling have been found to play an important role in human cancers and that aberrant activation of this signaling pathway was observed in pancreatic tumors [2–4]. Direct targeting of β-catenin and its functional partners (LEF/TCF proteins) by chemo preventive agents attracted attention in cancer therapeutics [34]. In the present study, we investigated a novel mechanism by which capsaicin inhibits the proliferation of pancreatic cancer cells. Our current results demonstrated that capsaicin treatment inhibits β-catenin-TCF-1 signaling, thereby decrease downstream transcriptional responsive genes cyclinD1 and c-Myc, which act to promote cell cycle and cell proliferation. Cleavage of caspase-3 was also observed after capsaicin treatment indicating occurrence of apoptosis. Our results further revealed that capsaicin treatment inhibits nuclear localization of β-catenin and TCF-1 and therefore disrupts nuclear β-catenin/TCF-1 complex, which is important for the transcription of cell survival genes. Our results also demonstrated that STAT-3 orchestrates β-catenin-TCF-1 signaling, which is inhibited by capsaicin. In addition, capsaicin mediated pancreatic tumor growth suppression was associated with the inhibition of β-catenin-TCF-1 signaling *in vivo*, consistent with our *in vitro* data.

Previous studies have demonstrated that inhibition of oncogene β-catenin by small molecules prevents the growth of esophageal and colon carcinoma cells [17, 35]. In agreement, our current study also showed that capsaicin treatment inhibits the activation of dishevelled family protein DvI-1. This in turn activated APC/Axin/GSK-3β complex, increased the phosphorylation of β-catenin, and inhibited TCF-1/β-catenin mediated transcription of responsive genes such as c-Myc and Cyclin D1. Recent studies also concluded that activated Wnt signaling inactivates GSK-3β activity, leading to accumulation of cytoplasmic β-catenin and induction of TCF-1/β-catenin mediated downstream target genes such as c-Myc, cyclin D1 [36–40]. Another study reported that nonsteroidal anti-inflammatory drugs (NSAIDS) inhibit TCF-1/β-catenin mediated downstream target genes such as cyclin D1 and thereby inhibits growth of colorectal cancer cells [41]. Our results validated such reports as capsaicin mediated inhibition of β-catenin and TCF-1 signal further inhibited c-Myc and cyclin D1, leading to apoptosis in pancreatic cancer cells. Recent studies on structural elucidation of β-catenin/TCF complexes highlight the possibility of developing cancer drugs that may disrupt this typically large hydrophobic interface of interacting proteins. A modest disruption of the binding equilibrium could establish value in treating cancer or other diseases [42–44]. Our present study indicates that capsaicin disrupts the interaction of β-catenin/TCF complex in the nucleus of pancreatic cancer cells and thereby suppresses β-catenin/TCF mediated cell proliferative genes such as c-Myc and cyclin D1.

Mutation in the multiple components of Wnt pathway including APC, Axin and β-catenin can result in increased levels of β-catenin [5, 16, 45, 46]. This may be due to mutational changes in GSK-3β phosphorylation site, preventing β-catenin phosphorylation and its proteosomal degration. Capsaicin treatment in the current study increased β-catenin degradation and enhanced its proteasomal degradation. Recent findings reported that lithium chloride and SB415286, a Wnt signaling activation mimic inhibits GSK-3β, which functions in the degradation of free β-catenin [47, 48]. Activation of Wnt signaling stabilizes cytosolic β-catenin, which translocate to the nucleus, stimulates TCF/LEF transcription factor protein and target transcription responsive genes such as cyclin D1 and c-Myc to promote cell proliferation [49, 50]. Our study demonstrated that capsaicin treatment also inhibits β-catenin/TCF-1 signaling activated by lithium chloride or SB415286.

**Figure 7:**
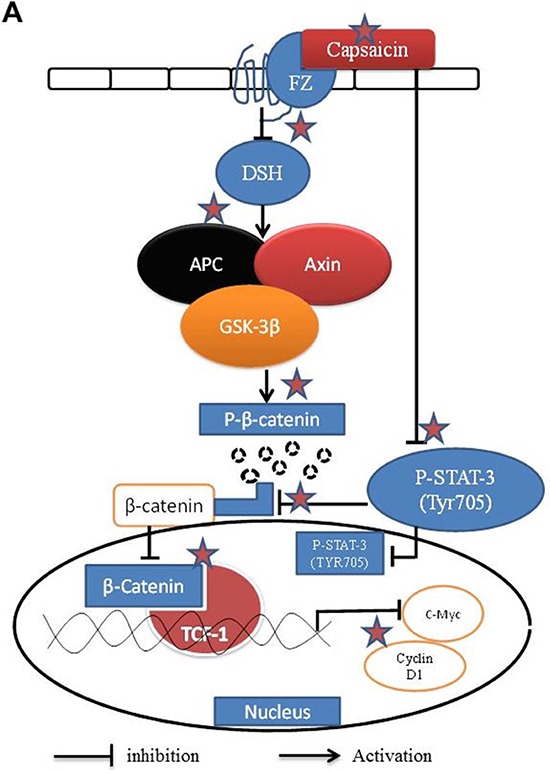
Schematic mechanism of action of capsaicin in pancreatic cancer cells targeting β-catenin pathway ☆Preferential sites of action by capsaicin.

Signal transducer and activator of transcription 3 (STAT-3) is as a member of the STAT family which participates in normal cellular responses as transcription factor [50]. Several previous studies reported that STAT-3 is a target of β-catenin/TCF-1 signaling [51, 52]. Interestingly few other studies also reported that activated STAT-3 was involved in β-catenin nuclear accumulation in human colorectal tumors [20]. Our current and previous study indicated that STAT-3 is up regulated in pancreatic cancer cells [53]. We found that capsaicin treatment decreased the translocation of β-catenin, TCF-1 and phosphorylation of STAT-3 (Tyr 705) in the nucleus. Our present findings also showed that STAT-3 over-expression increased β-catenin, TCF-1 and further downstream molecules cylin D1 and c-Myc levels, which were inhibited by capsaicin treatment. Our results are in agreement with the studies, which suggest that STAT-3 regulates β-catenin/TCF-1 signaling to promote pancreatic tumor progression. As a proof-of-concept, oral administration of 5 mg/kg capsaicin suppressed the growth of orthotopic pancreatic tumors in athymic nude mice [32]. Tumors from capsaicin treated mice showed decreased β-catenin, TCF-1 and p-STAT-3 levels as evaluated by immunofluorescence and western blotting. Capsaicin treated tumors also showed cleavage of caspase-3 indicating apoptosis. The mechanism of action of capsaicin targeting β-catenin signaling is shown in Figure 7. Overall our results demonstrated that capsaicin mediated inhibition of β-catenin/TCF-1 signaling results in apoptosis which was orchestrated by STAT-3 in pancreatic cancer cells *in vitro* and *in vivo*.

## MATERIALS AND METHODS

### Chemicals and antibodies

Capsaicin (purity > 99%), anti-actin, LiCl and SB415286 were obtained from Sigma (St. Louis, MO). The antibodies against Cl-caspase-3, Frizzled, DVI-1, p-β-Catenin (S33/37/T41), β-Catenin, p-GSK-3β (S9), GSK-3β, APC, Axin, p-STAT-3 (Tyr 705), STAT-3, TCF-1, c-Myc, Cyclin D1 were purchased from Cell Signaling (Danvers, MA). Lamin B was purchased from Santa Cruz Biotechnology, Inc (CA). Alexa fluor 488 (green), alexa fluor 594 (red) conjugated goat- anti-rabbit secondary antibody and DAPI were obtained from Invitrogen. MG-132 was procured from Calbiochem (San Diego, CA). AG-490, a specific inhibitor of STAT-3 was purchased from Sellack Chemicals LLC.

### Cell culture

Human pancreatic cancer cell lines PanC-1, L3.6PL and MiaPaCa-2 were obtained from ATCC (Rockville, MD). Monolayer cultures of PanC-1 and MiaPaca-2 cells were maintained in DMEM medium and L3.6PL was maintained in MEM medium, supplemented with 10% fetal bovine serum, PSN antibiotic mixture (10 ml/l), 2 mM L-glutamine, 10 mM HEPES, 1 mM sodium pyruvate and 20% glucose. All the cultures were maintained at 37°C in a humidified chamber of 95% air and 5% CO_2_. Cell lines were verified by STR analysis at TTUHSC core facilities (Lubbock, TX). All the three cell lines have mutant KRAS and TP53. Panc-1 has homozygous deletions in CDKN2A and p16 genes and wild type SMAD4. MiaPaCa-2 has homozygous deletion of p16 gene. L3.6PL exhibits homozygous deletion of SMAD4.

### Transient transfection

PanC-1 cells were transiently transfected with STAT-3 plasmid using Nucleofector transfection reagent (Lonza, Walkersville, MD) according to the manufacture instruction. Briefly 1 × 10^6^ PanC-1 cells were collected and suspended in 100 μl of nucleofection solution per sample and then combined with 2 μg STAT-3 DNA. Once the electroporator program was completed, 500 μl of pre-incubated respective medium was added to the cuvette and samples were transferred into the 6well plate for transfection. Transfected cells were treated with 75 μM capsaicin for indicated time periods.

### MG132, AG-490, LiCl, SB415286 and IL-6 treatment

In separate experiments, pancreatic cancer cells were treated with either 10 μM MG132, 20 μM AG-490, 40 mM LiCl or 50 μM SB415286 for 1 h or with 20 ng/ml IL-6 for 15min, followed by treatment with 75 μM capsaicin for 24 h. Cell lysate were prepared for western blotting as we described previously [32].

### Immunoprecipitation assay

To examine the effect of capsaicin on the nuclear interaction of β-catenin with TCF-1 protein, immunoprecipitaion assay was performed as described by us previously [32]. Briefly, PanC-1 cells were treated with DMSO or 75 μM capsaicin for 24 h, nuclear fraction was separated using a nuclear fractionation kit and immunoprecipitated with β-catenin or TCF-1 antibodies. Samples were immunobloted with β-catenin or TCF-1 antibodies.

### Immunofluorescence assay in cells and tumor section

Immunofluorescence assay was performed as described by us previously [54]. Briefly, PanC-1 cells were plated on coverslips and allowed to attach overnight and then treated with 75 μM of capsaicin for 24 h. On the other hand, at the end of the orthotopic experiment, tumors from control and capsaicin treated mice were dissected out. About 10 μm tumor sections were immediately sliced, placed on glass slides and kept frozen at –80°C for 24 h. Treated and untreated cells and tumor sections were fixed with acetone:methanol (1:1) mixture and blocked with goat-serum for 1 h and incubated with β-catenin, TCF-1 and p-STAT-3(Tyr705) antibodies overnight at 4°C. Immunofluoresence was detected by anti-rabbit immunoglobulin G (IgG) conjugated with alexa fluor 594 (Invitrogen) (red), alexa fluor 488 (green) and DAPI (blue). After four washings, coverslips were mounted with antifade mounting reagents. Nuclei were stained with DAPI and the immmunofluoresence was observed by a fluorescence microscope using oil immersion at 60X magnification.

### *In vivo* orthotopic experiment

Pancreatic orthotopic implantation in nude mice was performed previously, and the tumors from control and capsaicin treated mice were used in this study [32]. All animal experiments proceeded according to the Institutional Animal Care and Use Committee (IACUC). Briefly, 4–6 week old female athymic nude mice were anesthetized and a small incision was made to implant stably luciferase-expressing 1 × 10^6^ PanC-1 (PanC-1-luc) cells into the sub-capsular region on the pancreas. Skin incisions were then closed. On the very next day of surgery, animals were imaged for basal luminescence using IVIS Bio Luminescent System equipped with Living Image software (Caliper LifeSciences, MA) after injecting luciferin (3 mg/mouse). Twenty-one days after the surgical implantation of PanC-1-luc cells, mice were randomly divided into two groups with 5mice in each group. Group I served as controls and received 0.1 ml vehicle every day by oral gavage. Group II received 5 mg capsaicin/kg of body weight every day by oral gavage. Tumor luminescence and animal weight was measured thrice a week for seven weeks. At the end of the experiment (day 70), mice were sacrificed; tumors and pancreas were excised from each mouse, weighed and snap frozen.

### Western blot analysis

Cells were exposed to various concentrations of capsaicin for different time points and lysed on ice as described by us previously [32, 53]. Whole-cell extracts were also prepared as mentioned above. The tumors from control and capsaicin treated mice were minced and lysed by the described procedure [32]. The cell lysate was then cleared by centrifugation at 14,000 g for 30 min. Cell lysate containing 10–80 μg protein was resolved by 6–12.5% sodium dodecyl sulfate-polyacrylamide gel electrophoresis (SDS-PAGE) and the proteins were transferred onto polyvinylidene fluoride membrane. After blocking with 5% non-fat dry milk in Tris buffered saline, the membrane was incubated with desired primary antibody (1:1000 dilutions) overnight. Subsequently, the membrane was incubated with appropriate secondary antibody (1:2000 dilutions) and antibody binding was detected by using enhanced chemiluminescence kit, according to the manufacturer's instructions. Each membrane was stripped and re-probed with antibody against actin (1:20000 dilutions) to ensure equal protein loading.

### Statistical analysis

All statistical calculations were performed using Graph Pad Prizm 5.0. Analysis of variance (ANOVA) was used to test the statistical significance of difference between control and treated groups followed by Bonferroni's post-hoc analysis for multiple comparisons. *P*-values less than 0.05 were considered statistically significant.
